# A Comprehensive Outlook on Dilated Cardiomyopathy (DCM): State-Of-The-Art Developments with Special Emphasis on OMICS-Based Approaches

**DOI:** 10.3390/jcdd9060174

**Published:** 2022-06-01

**Authors:** Vivek Sarohi, Shriya Srivastava, Trayambak Basak

**Affiliations:** 1 Indian Institute of Technology (IIT)-Mandi, School of Basic Sciences (SBS), Mandi 175075, HP, India; d19056@students.iitmandi.ac.in (V.S.); 1995shriya@gmail.com (S.S.); 2 BioX Centre, Indian Institute of Technology (IIT)-Mandi, Mandi 175075, HP, India

**Keywords:** dilated cardiomyopathy (DCM), heart failure, gene, biomarkers, mechanism, treatment, proteomics, lipidomics

## Abstract

Dilated cardiomyopathy (DCM) remains an enigmatic cardiovascular disease (CVD) condition characterized by contractile dysfunction of the myocardium due to dilation of the ventricles. DCM is one of the major forms of CVD contributing to heart failure. Dilation of the left or both ventricles with systolic dysfunction, not explained by known causes, is a hallmark of DCM. Progression of DCM leads to heart failure. Genetic and various other factors greatly contribute to the development of DCM, but the etiology has still remained elusive in a large number of cases. A significant number of studies have been carried out to identify the genetic causes of DCM. These candidate-gene studies revealed that mutations in the genes of the fibrous, cytoskeletal, and sarcomeric proteins of cardiomyocytes result in the development of DCM. However, a significant proportion of DCM patients are idiopathic in nature. In this review, we holistically described the symptoms, causes (in adults and newborns), genetic basis, and mechanistic progression of DCM. Further, we also summarized the state-of-the-art diagnosis, available biomarkers, treatments, and ongoing clinical trials of potential drug regimens. DCM-mediated heart failure is on the rise worldwide including in India. The discovery of biomarkers with a better prognostic value is the need of the hour for better management of DCM-mediated heart failure patients. With the advent of next-generation omics-based technologies, it is now possible to probe systems-level alterations in DCM patients pertaining to the identification of novel proteomic and lipidomic biomarkers. Here, we also highlight the onset of a systems-level study in Indian DCM patients by applying state-of-the-art mass-spectrometry-based “clinical proteomics” and “clinical lipidomics”.

## 1. Introduction

Dilated cardiomyopathy (DCM) is characterized as a disorder of the heart muscle. It is distinguished by the widening/dilation of the left ventricle of the heart with left ventricular or biventricular systolic dysfunction [1,2]. Contractile functioning of the left ventricle is highly compromised due to dilation. However, in some cases of DCM, the right ventricle is also dilated. Dilation of the ventricle leads to progressive disease resulting in heart failure. DCM is positioned third on the list of the main causes of heart failure [3,4], and it is most prevalent among all forms of cardiomyopathies in children [5]. Dilation and dysfunction in the contractility of the left ventricle can be caused by several pathological conditions including viral infection, drug abuse, toxins, and autoimmune disorders. However, around 50% of cases of DCM are etiologically unknown [6]. These etiologically unknown cases are termed as idiopathic DCM. In many cases of DCM, the early clinical symptoms are not highly observable and some patients are asymptomatic. The symptoms of DCM can be intermittently observed at the initial phase of DCM for certain patients. These clinical symptoms (Table 1) become more pronounced at the advanced (severe) stage of DCM [7,8,9,10]. Genetically inherited defects are a major cause of DCM. Around 30–35% of idiopathic cardiomyopathies are caused by genetic defects [10]. The very first attempt to assess the prevalence of DCM (36.5 in 100,000) was performed by Codd et al. during 1975–1984 in the Olmsted Country population (Minnesota, USA). Males were found more prone to DCM than females with a prevalence ratio of 3:1 [11]. In Italy, 2 years of data (1987–1989) of postmortem study in the Department of Pathology at Trieste University estimated the prevalence of DCM to be 4.5 in 100,000 subjects per year, and clinical data showed the prevalence of 2.5 in 100,000 subjects. However, combining DCM causing mortality, the total prevalence was found to be 6.95 in 100,000 subjects [12]. In the last 30–40 years, many epidemiological studies have been undertaken on DCM. However comprehensive data about the incidence of DCM globally is not known but the estimate of prevalence is >1 out of every 250–400 persons [13,14].

**Figure 1:**
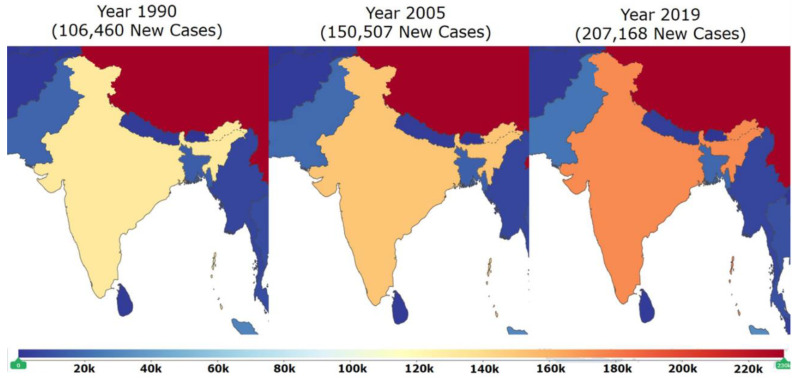
This map of India (modified from Global Burden of Disease; GBD) shows the increase in the incidence of DCM in India in years between 1990 to 2019. There were 106,460 new cases in 1990. Incidents massively increased and 150,507 new cases were reported in 2005. Spike in incidence increased further and 207,168 new cases were reported in 2019. This data clearly indicates that the incidence of DCM is increasing at an alarming rate in India [Adapted with permission from IHME, Seattle, DC, USA “Source: Institute for Health Metrics Evaluation. Used with permission. All rights reserved” [15]. Accessed on 18 April 2021.

The prevalence of DCM has not been well studied in India compared to many western countries in the world. However, the global burden of disease [15] data shows that there is a drastic increase in the incidence of DCM in India. There were 106,460 new cases of DCM in 1990 in India. In one and a half decades, incidence increased massively and 150,507 new cases were reported in the year 2005. A spike in the incidence is consistent because 207,168 new cases were reported in the year 2019 [15]. This data clearly indicates the massive increase in the incidence of DCM in the past three decades (Figure 1) in India. Although, it is difficult to ascertain whether this increase in the incidence of DCM is highlighted because of the advanced detection systems [16], but the increasing incidence of DCM in India is alarming and has emerged as a major public health problem in the current scenario. There is the utmost need for better early diagnostic, prognostic biomarkers, effective treatments, and optimized preventive strategies to combat this disease. In this comprehensive review of DCM, we summarize the symptoms, causes, mechanistic insights of DCM development, available state-of-the-art diagnosis, and treatments, with emphasis on potential novel therapies and ongoing clinical trials. Further, we also showcase the importance of identifying a newer systems-level biomarker panel for DCM patients in India using next-generation proteomic- and lipidomic-based methods.

## 2. Causes of DCM

### 2.1. Causes of DCM in Children and Newborns

#### 2.1.1. Myocarditis

Virus, allergens, toxins and autoimmune responses are responsible for myocardial damage [17]. This damage, caused by inflammation in general, is evidenced by the infiltration of immune cells into the myocardium associated with the degeneration and necrosis of myocytes during myocarditis. Importantly, histological evidence along with a specific threshold of leucocytes, monocytes and T-lymphocytes defines the myocarditis status [17,18,19,20]. Viral myocarditis is a common cause of DCM. Coxsackie B virus infection has a high tendency to lead to DCM. Coxsackie B is categorized under the picornavirus family and enterovirus genus. It also has a close association with echovirus, poliovirus, and rhinovirus. Other viruses are also found to contribute to DCM. Coxsackie (A, B), Herpes simplex, Epstein-Barr virus, Rubeola, Coronavirus, Adenovirus, Cytomegalovirus, Mumps, Vaccinia, Hepatitis B, Influenza (A, B), Varicella-zoster, Rubella, HIV and rabies viruses cause myocarditis that leads to the DCM [21].

#### 2.1.2. Selenium Deficiency

Selenium is one of the trace elements present in animal bodies. Selenium is an important element of selenoproteins which play a crucial role in various endocrine, immune, and reproductive processes. Glutathione peroxidases (*GSH-Px*) is a selenoprotein that inactivates peroxidase. Selenium is useful in cardiomyocytes for the function of the antioxidant defense mechanism and a deficiency of selenium can lead to DCM [22].

#### 2.1.3. Malformation in Pulmonary Arteriovenous

Pulmonary arteriovenous malformation (PAVM) is a condition of anomalous communication between veins and pulmonary arteries. Congenital pulmonary arteriovenous malformations have a close relation with hereditary hemorrhage telangiectasia. The shunting of blood from the pulmonary artery to veins causes the delivery of low oxygen to the heart. Shunting of blood causes hypoxia which leads to pulmonary hypertension and imparts a high load on the left ventricle resulting in DCM causing a reduced ejection fraction [23]. However, congenital heart diseases should be diagnosed to understand the overlapping contributory phenotypes of the DCM pathology in patients.

#### 2.1.4. Endocardial Fibroelastosis

Endocardial fibroelastosis is caused by the deposition of collagen and fibroelastin fibers in the endocardium. The mitral valve leaflet is thickened, and the left ventricle is dilated in endocardial fibroelastin. Over a period of time, it develops into DCM [24].

#### 2.1.5. Noncompacted Myocardium

Noncompacted myocardium is associated with the regional thickness of the ventricular wall and it can lead to heart failure [25]. Mutations in the protein-encoding genes of tafazzin, lamin, actin, titin and others are involved in non-compact myocardium disease. These gene mutations eventually lead to DCM and heart failure.

#### 2.1.6. Calcium Deficiency

Calcium deficiency causes irreversible DCM in infants. Calcium is essential for myocardial contraction. A deficiency of calcium in infants affects myocardial contractility [26].

#### 2.1.7. Idiopathic DCM (IDCM)

IDCM contributes to 50 to 70% of the total number of cases of DCM in children [5]. Many cases of IDCM are assumed to have genetic causes. In a number of cases, IDCM is reclassified as familial DCM after family investigation and testing. There is no specific observable characteristic difference present between familial DCM and idiopathic DCM [5].

#### 2.1.8. Barth Syndrome

Barth syndrome is a heart muscle syndrome caused by mutation in the tazzafin protein-encoding TAZ (*G4.5*) gene [27]. It is mostly present in males and it is often present from birth. It is characterized by muscle dilation and skeletal myopathy. In Barth syndrome, the heart muscles fail to perform the movement function properly. Children with Barth syndrome find it hard to move or crawl and they feel very tired even after little amount of effort. In many cases, Barth syndrome goes unnoticed in the early life of a patient. The development of noticeable symptoms varies greatly from person to person [28].

#### 2.1.9. Familial/Genetic Pediatric DCM

Genetic causes are one of the major reasons for the occurrence of DCM. There are similarities in the genetic causes of newborns and adults. Rampersaud et al. [29] studied 41 cases of pediatric DCM. They identified 15 genes associated with DCM pediatric patients. Out of these 15 genes, 9 genes (*MYH7*, *SCN5A*, *TNNT2*, *LMNA*, *MYBPC3*, *MYH6*, *TNNC1*, *TNNI3* and *TPM1*) are also known to cause DCM in adults. Recently, Khan et al. [30] did a cohort study on 109 pediatric patients and found that *TTN* (Titin) truncation was the most prevalent cause of genetic DCM in pediatric patients. Along with *TTN*tv, genetic variations (mutation) were also detected in *MYH7* among pediatric DCM patients.

### 2.2. Causes of DCM in Adults/Adolescents

#### 2.2.1. Familial/Genetic

It is estimated that 30–50% of DCM cases fall under this category [31]. DCM is highly linked with autosomal dominant inheritance, but X-linked and autosomal recessive inheritance has also been found to be associated with DCM. Mutations in the cytoskeletal and sarcomeric genes of cardiomyocytes are considered to be responsible for DCM [31]. Cytoskeletal genes including *TTN* (titin), *DES* (desmin), *LMNA* (lamin A/C), *ABLIM1* (actin binding LIM domain protein), *ACTN2* (α-actinin-2), *NEBL* (nebulette), *MYPN* (myopalladin), *SGCD* (δ sarcoglycan) and *ZASP* (Z band alternatively spliced PDZ domain protein) are involved in DCM [32]. Mutations in sarcomeric genes including *ACTC1* (cardiac actin alpha), *TNNT2*, *TNNI3*, *TNNC1* (troponin T2, I3 and C1), *MHY7* (β- myosin heavy chain), *TPM1* (tropomyosin-1), *PLN* (phospholamban), *MYBPC3* (myosin binding protein C) and *SCN5A* (Sodium channel protein type 5 subunit alpha) are associated with DCM (Table 2).

#### 2.2.2. Alcohol

A daily consumption of alcohol of more than 35.2 mL in males and 17.6 mL in females can cause alcohol cardiomyopathy [35]. Although it remains debatable that a low consumption of alcohol can be useful in coronary artery disease (CAD), hemorrhagic stroke and heart failure, alcohol abuse increases blood pressure and adversely affects the immune system, which increases the chances of myocarditis [35]. Carbohydrate-deficient transferrin and elevated levels of liver enzyme with cardiomyopathy are diagnostic measures of alcohol-induced cardiomyopathy [35].

#### 2.2.3. Myocarditis

Myocarditis affects both at early age and adult age due to several virus-mediated infections. The mode of action of myocarditis remains the same at all stages of life.

#### 2.2.4. Tachycardiomyopathy

Tachycardiomyopathy, also known as tachycardia-induced cardiomyopathy, is a left ventricle disorder. It is a cardiac condition which can be partially or completely reversed. If tachyarrhythmia is controlled then tachycardiomyopathy can also be reversed to normal stage [36].

#### 2.2.5. Mitochondrial Diseases

Mitochondrial disorders including NADH-coenzyme Q reductase (complex I) deficiency, deficiency of cytochrome C oxidase, lactic acidosis, myoclonic epilepsy with ragged red fibers (MERFF), Kearns–Sayre syndrome, encephalopathy associated with mitochondria, and most importantly carnitine palmitoyl transferase II deficiency, significantly contribute to developing DCM [37].

#### 2.2.6. Cardiomyopathy Associated with Right Ventricular Arrhythmia

Although less prevalent, it is a life-threatening condition entailing sudden death in young and sports persons. Mutation of the desmosomal protein gene leads to this disease which is now considered as genetic myocardial dystrophy [38].

#### 2.2.7. Eosinophilic Myocarditis

Eosinophilic granulomatosis with polyangiitis (EGPA), also known as Churg-Strauss syndrome, is characterized by systemic vasculitis. This vasculitis affects the small and medium sized blood vessels [39]. The effects of this disease in the heart are dangerous and can lead to heart failure [40]. Eosinophilic myocarditis is diagnosed with left ventricle enlargement and decreased systolic function. If eosinophilic myocarditis is caused by Churg-Strauss syndrome then treatment of the Churg-Strauss syndrome can reverse the eosinophilic myocarditis to the normal stage [41].

#### 2.2.8. Toxins

Over-exposure to heavy metal toxins can lead to DCM. Lead (Pb), cobalt (Co) and arsenic (As) over-intoxication can lead to DCM. Iron overload is also found to cause DCM [42]. Under certain genetic and metabolic disorders, the amount of circulating iron exceeds the handling capacity of transferrin. Free, unbound iron is a potent free radical which causes oxidative damage to the cardiomyocytes. Lead is a toxin which attacks the cardiovascular system adversely. Cobalt is useful in the production of red blood cells, but a very high amount of blood cells due to a higher level of cobalt can affect the heart rate by interfering with calcium binding to sarcolemma [42]. Thus, it causes trouble in contractility in the heart. Arsenic is a toxin which directly attacks cardiac muscles. It dilates cardiac capillaries and it induces DCM [42].

#### 2.2.9. Peripartum Cardiomyopathy

It is a life-threatening disease. It is developed during pregnancy in females. It severely affects females during late pregnancy or the puerperium phase. In peripartum DCM, cardiac failure can be observed towards the end of the gestation period to the 5-month postpartum period [43].

#### 2.2.10. Endocrinopathy

A hormonal imbalance in the body can lead to the development of DCM. An imbalance of the thyroid hormones leads to heart disorders. Tachycardia and palpitations are common in patients with thyroid hormone imbalance [44]. The catecholamine secretion from tumors induces cardiac remodeling [45]. A low level of insulin causing persistent hyperglycemia severely compromises cardiac functions leading to DCM or other cardiomyopathies [46].

#### 2.2.11. Nutritional Deficiency

Low levels of thiamine (B_6_), carnitine deficiency and hypophosphatemia have a correlation with DCM progression. Nutritional deficiency leads to the abnormal functioning of cardiac muscles and long-term nutritional deficiencies can lead to DCM and other disorders [47].

## 3. Genes Involved in Molecular Mechanisms of DCM

**Figure 2:**
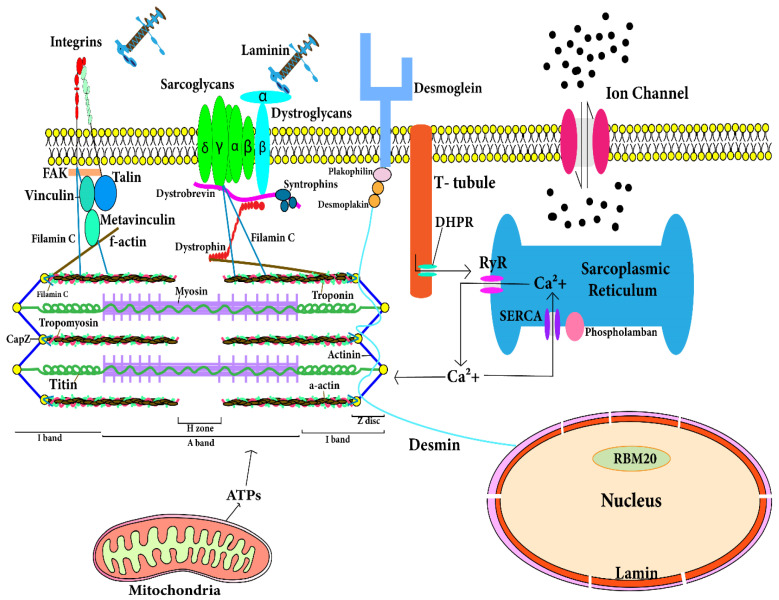
Location of proteins in cardiomyocytes that are responsible for development of dilated cardiomyopathy. The figure depicts the spatial location of the cytosolic and sarcomeric proteins encoded by corresponding genes. Mutations in these genes contribute to development of dilated cardiomyopathy. The T-tubule has a large number of ion channels and transporters. T-tubule regulates the calcium concentration in cardiomyocytes and transmits the action potential into the cardiomyocyte. RyR (ryanodine receptor) in sarcoplasmic reticulum (SR) of cardiomyocyte interacts with T-tubule for action potential transmission and RyR is responsible for release of stored calcium from SR. Calcium and ATPs are utilized by sarcomere in contraction. After contraction, calcium released from sarcomere is stored in SR through SERCA. Sarcolemmal and cytoskeletal proteins maintain architecture and functions of cardiomyocytes. Mutation in any of these protein-encoding genes leads to dilated cardiomyopathy.

Cardiomyocytes are comprised of fibrous proteins, cytoskeletal proteins, and sarcomeres. Mutations in the following genes encoding the respective proteins lead to the development of DCM. A comprehensive spatial map of such structural proteins is depicted in Figure 2. Following are the genes which significantly contribute to the development of DCM.

### 3.1. Troponin T Gene (TNNT2)

Troponin T protein of the thin sarcomere filament is encoded by *TNNT2* gene. *TNNT2* is 17 exons containing 17 kb long genomic DNA. Alternative splicing of this gene produces different isoforms of troponin T. Some 288 amino acids are found in the primary isoform of troponin T and it has two major domains. Tropomyosin interacts with the NH_2_ terminal domain of troponin T, troponin C and troponin I interact with the COOH terminal domain of troponin T. Deletion (Lys210) in the *TNNT2* gene is found in DCM [48]. In DCM, deletion in *TNNT2* gene is found on the carboxyl domain of 207–234 residues which communicate with calcium-sensitive troponin C. Thus, this deletion contributes to a calcium imbalance in cardiomyocytes and their misfunction.

### 3.2. Cardiac Actin Alpha Gene (ACTC1)

Actin, a protein of the thin sarcomere filament is encoded by the *ACTC1* gene. Out of 20 genes associated with actin which are present in the human genome, four genes code for smooth muscle actins, skeletal actins and cardiac actins. Actin corresponding to cardiac and skeletal tissues are present in the heart, but skeletal actin has a lesser amount in the adult human [49]. The cardiac actin gene (*ACTC1*) encodes 375 amino acids which are coded by six exons. The myosin cross-bridge attaches to the NH_2_ terminal domain of the cardiac actin protein. Further, α-actinin and dystrophin bind with the COOH terminal domain of *ACTC1*. *ACTC1* participates mainly in the contraction of muscles. This gene was found to be involved in autosomal dominant DCM in adults [49]. Two missense mutations are found in the *ACTC1* gene present in DCM patients [49]. Mutation in the cardiac actin at the juncture of the Z line is found in DCM. This immobilized end of actin in the Z line is believed to be associated in force transfer to the extra-sarcomeric cytoskeleton via the sarcomere.

### 3.3. Myosin Heavy Chain—β Gene (MYH7)

The protein β-myosin heavy chain of the thick sarcomere filament is encoded by the *MYH7* gene. The myosin protein is present in hexameric form with two heavy chains and four light chains. β-MHC and α-MHC are two isoforms of the myosin heavy chain protein present in cardiac myocytes. The β-myosin heavy chain is predominantly expressed in the atrium and ventricles of embryos or some skeletal muscles. However, in adults, the α-MHC (encoded by *MYHC6*) predominates. *MYHC7* is a 23 kb long DNA. Out of 41, 38 exons make up a 1935 amino acid long protein product. Two missense alterations associated with the *MYH7* gene have been found to significantly contribute to the development of DCM [48]. First is the *MYHC7* (Ser532Pro) mutation which affects α-helical of the lower domain of the myosin heavy chain. Mutation in this domain disrupts the actin–myosin binding. The second missense mutation is the *MYH7* (Phe764Leu) mutation. It affects the hinge region and it adversely affects contraction by changing the polarity of the cross-bridge movement.

### 3.4. Cardiac Myosin Binding Protein C Gene (MYPBC3)

Cardiac myosin binding protein C (cMBP-C) is a thick filamented protein that is encoded by the *MYPBC3* gene. This cMBP-C is the exclusive isoform present in the A band of sarcomere, and it forms 7 to 9 transverse bands with a spacing of 43 nm. The *MYPBC3* gene is made up of 24 kb of DNA. The 37 exons of the *MYPBC3* gene encode 1274 amino acids. This protein has multiple sites including a phosphorylation region, fibronectin type 3 domain, a cardiac-specific region and intervening titin and myosin binding sites. Missense mutation in this gene is found in DCM patients [50].

### 3.5. Tropomyosin α Gene (TPM1)

Its protein product can be found in fast skeletal muscle or ventricular myocardium. Fifteen exons are present in *TPM1* gene. Five exons are constitutively present in all isoforms and alternating splicing of the other 10 exons gives rise to different isoforms of the protein. Some 288 amino acids are encoded by 10 exons of cardiac α-tropomyosin isoform. α-tropomyosin contains Ca^2+^ sensitive and Ca^2+^ insensitive binding sites. Two missense mutations in the *TPM1* gene are found in individuals having DCM [51]. These two mutations are believed to be responsible for change in actin interactions and the structural integrity of α-tropomyosin.

### 3.6. Titin Gene (TTN)

Giant sarcomeric protein titin is encoded by the *TTN* gene. Titin (3816 kDa) is the largest known polypeptide. Titin has a stiffer portion within the A band and an extensible portion within the I band. The repeating 298 immunoglobulin domain and fibronectin 3 domain contribute 90% of the total mass of titin. The elasticity and organization of sarcomere is maintained by titin. Frameshift and missense mutation in the *TTN* gene are found in individuals having DCM [52]. Mutations resulting in titin truncation variants (TTNtv) contribute to almost 25% of genetic cause in idiopathic DCM and almost 18% is estimated for sporadic cases [53]. Verdonschot et al. [54] studied 689 DCM patients and found *TTN*tv were most abundant in the patients with DCM. This study evidenced that the titin truncation contributes the most to familial/genetic DCM.

### 3.7. Desmin Gene (DES)

Desmin, generally considered a cytoskeletal protein of intermediate filament is encoded by the *DES* gene. Desmin is present in all muscle tissues. It is believed that it regulates myogenic differentiation and the desmin intermediate filament significantly contributes to force transmission from the sarcomere [55]. The *DES* gene is made up of 8.4 kb of genomic material. The desmin protein has 470 amino acids encoded by nine exons of the DES gene. Desmin tail domain missense mutation (Ile451Met) disrupts myofibril organization, and it is reported to significantly contribute to the development of DCM [55]. Desmin knockout in mouse models also develops DCM [55].

### 3.8. δ-Sarcoglycan Gene (SGCD)

δ-sarcoglycan glycoprotein of transmembrane of sarcolemma is encoded by the *SGCD* gene. Some sarcoglycan proteins (α, β, γ and δ-sarcoglycan) form the sarcoglycan complex, which is a part of a glycoprotein transmembrane complex associated with dystrophin. The *SGCD* gene is active in striated and smooth muscles. The highest levels of the *SGCD* gene are expressed in cardiac and skeletal muscle. The *SGCD* gene is composed of around 100 kb of genomic DNA. δ-sarcoglycan has 290 amino acids encoded by eight exons of the *SGCD* gene. The δ-sarcoglycan protein has a large extracellular COOH terminal domain, a hydrophobic transmembrane domain, and a small intracellular domain. One missense alteration of the *SGCD* gene is associated with DCM and early unprecedented death [56]. Two other mutations in the *SGCD* gene leading to codon deletion, i.e., Lys238 codon deletion, are found in individuals with DCM [56].

### 3.9. Vinculin and Metavinculin Gene (VCL)

Metavinculin and vinculin are cytoskeletal proteins encoded by alternative splicing of the *VCL* gene. Metavinculin and vinculin are coexpressed in smooth, skeletal and cardiac muscle. α-Actinin, talin, and γ-actin interact with metavinculin and vinculin at subsarcolemmal costameres and they form a micro-filamentous network. This network links sarcolemma via the cytoskeleton. Metavinculin along with vinculin are seen on the intercalating disc of the adherens junction and they actively participate in cell-to-cell adhesion. Over 75 kb of genomic DNA makes up the structure of the *VCL* gene. It has 22 exons that make the vinculin protein comprising 1066 amino acids and metavinculin protein comprising 1134 amino acids. Their multimers of four to six molecules have head and tail domains. The head domain interacts with talin and the tail domain interacts with γ-actin and paxillin. High levels of vinculin to 1.2 fold were found in explanted human hearts from individuals having DCM [57]. On the other hand, absence or low levels of metavinculin are also found in individuals having DCM [58].

### 3.10. Lamin A and Lamin C Gene (LMNA)

Lamin A and lamin C protein of intermediate filament are encoded by the *LMNA* gene. Lamin proteins are present in nuclear lamina, located on the internal side of nuclear membrane. Lamin A has four [4] transcripts (lamin A, C, Aδ10, and C2) made from alternative splicing of *LMNA* gene, while lamin B has two transcripts (lamin B1 and B2) made by *LMNB1* and *LMNB2* genes. A, B2 and C types of lamins are present in skeletal muscle and cardiac muscle. These are believed to contribute to the integrity of nuclear membrane; *LMNA* gene is made up of 2400 bp of DNA and 12 exons. Lamin A transcript has 664 amino acids while lamin C transcript has 562 amino acids. Lamin A and C proteins have an α-helical dimer rod domain, globular head domain (NH_2_-terminal) and tail (COOH-terminal) domains. *LMNA* mutations are prevalent in families of autosomal dominant DCM [59]. A total of 19 mutations in *LMNA* gene are found in relation with autosomal dominant DCM [59].

### 3.11. Dystrophin Gene (DMD)

Dystrophin is a massive cytoskeletal protein, which is coded by the *DMD* gene. It is present in high amounts in skeletal, smooth and cardiac muscle cells and also in low amounts in brain cells [60]. Dystrophin is a large 2.4 mb gene. The 3685 amino acids are encoded by 79 exons of the *DMD* gene. The *DMD* gene has different promoters expressed in different tissues. Alternative splicing of the *DMD* gene give rise to different isoforms of dystrophin protein. Dystrophin is a rod-shaped protein and is around 150 mm long in length. Dystrophin is believed to contribute to maintaining membrane stability, force transmission and intracellular organization. Mutation in the *DMD* gene is found to contribute to X-linked DCM-associated death within just 1–2 years after diagnosis [61]. Deletion, missense, insertion, inversion and point mutations in the *DMD* gene result in abnormalities of the dystrophin protein [62].

### 3.12. Tafazzin Gene (G4.5)

Tafazzin, a group of proteins present in abundance in cardiac and skeletal muscles is encoded by *G4.5* gene. The molecular functions of tafazzins have remained elusive. The *G4.5* gene is made up of around 1100 bp of genomic DNA. It has 11 exons whose alternative splicing produce 10 different transcripts. Mutations in *G4.4* are found to be associated with X-linked Barth syndrome which is characterized by infantile-onset DCM [63]. Missense mutations in exon 8 and exon 10 conserved regions are found in the individuals with X-linked DCM [63].

### 3.13. Phospholamban Gene (PLN)

Phospholamban, a membrane protein is encoded by the *PLN* gene. Phospholamban is made up of 52 amino acids. It is a key regulatory protein in cardiac diastolic function and Ca^2+^ cycling in sarcoplasmic reticulum. Mutations in the *PLN* gene result in an imbalance of calcium in the cells. Deletion of arginine 14 (PLN-R14Del) in the *PLN* gene is found in individuals having DCM [64]. Mutation in the *PLN* gene (R9C) leads to the development of DCM in mice [65].

### 3.14. Mitochondrial DNA

Mitochondrial inheritance is maternal in nature. Cardiomyocytes contain high numbers of mitochondria for the production of energy and contractile function. Mitochondria has 16.6 kb DNA, which encodes 13 proteins involved in the oxidative phosphorylation. Certain genetic alterations such as deletions or point mutations in mitochondrial DNA lead to mitochondrial abnormality [66]. Mitochondrial abnormality contributes to the development of the fatal infantile form of DCM [67].

### 3.15. Filamin C (FLNC)

Filamin C is a large 2725 amino acid long protein. It is encoded by the *FLNC* gene. It plays important role in the function of skeletal and cardiac muscles. Begay et al. [68] found filamin C truncations to be associated with arrhythmogenic dilated cardiomyopathy. Recently Aggarwal et al. [69] discussed that haploinsufficiency (*FLNC*^+/−^) can cause dilated cardiomyopathy. Studies carried out on filamin C revealed that mutations in *FLNC* can lead to cardiomyopathies.

## 4. Mechanistic Insights for DCM Development

**Figure 3:**
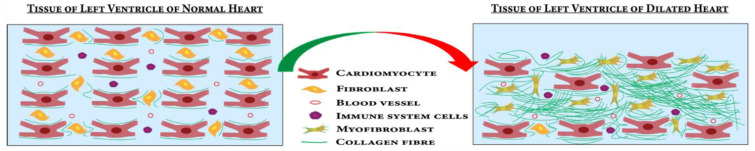
In DCM, cardiomyocytes undergo apoptosis and proliferation of cardiac fibroblasts to myofibroblasts causes fibrosis in the left ventricle of the heart. These processes lead to left ventricle dilation and wall thinning.

DCM progression is initiated by various genetic and idiopathic factors. Classically, “Frank-Starling law” described the causal relationship between resultant contraction force with cardiac fiber length [70]. Mutations in sarcomeric, non-sarcomeric and cytoskeletal protein components have been attributed mechanically to cardiac contraction reduction during DCM [33]. During the pathological progression of DCM, cardiomyocytes gradually undergo various changes leading to compromised function and morphology leading to reduced systolic performance with dilated ventricles [71]. In DCM, significant death of cardiomyocytes occurs by the process of apoptosis (Figure 3) [72]. Accumulation of collagens and other extracellular matrices (ECM) proteins in the ECM followed by the cardiomyocyte apoptosis leads to myocardial fibrosis [73]. The proliferation of cardiac fibroblasts to myofibroblasts in response to profibrotic stimuli contributes majorly to the fibrosis [74]. These processes lead to the dilation and stiffening of the left ventricle, a definitive hallmark of DCM [75] (Figure 3). The myocardium becomes thinner and the ventricle is stretched, leading to dysfunction in heart [76].

### 4.1. Signaling Pathways Involved in DCM

Cardiomyocyte death and fibrosis are two major processes that lead to the development of DCM. The following pathways involved in cell death and fibrosis during DCM are described below.

#### 4.1.1. Cell Death Pathways in Cardiomyocytes

**Figure 4:**
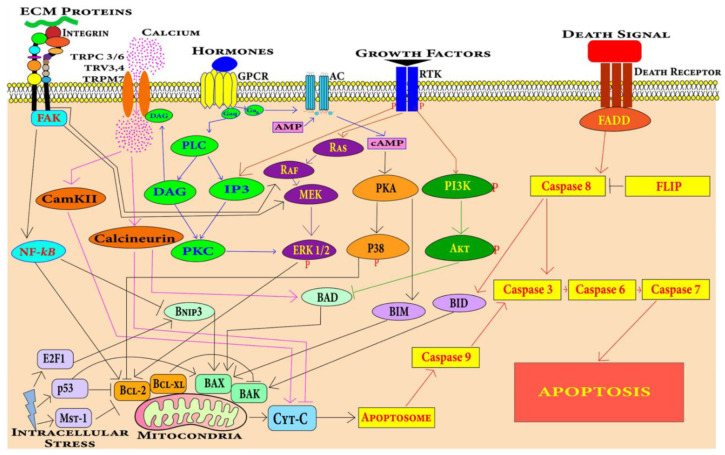
Cardiomyocytes can undergo apoptosis through extrinsic or intrinsic apoptosis pathways. Death receptors mediate the extrinsic apoptosis pathway and various factors including mechanical stress, oxidative stress, DNA damage, growth factors and hormones induce the intrinsic cardiomyocyte apoptosis pathway.

Apoptosis in cardiomyocytes can be induced by various stimuli including hypoxia, mechanical stress, oxidative stress, hormones, growth factors, death ligand including tumor necrosis factor, Fas ligand and anthracycline drugs [77,78]. Myocardium-specific apoptosis in the transgenic mouse model revealed that even low levels of myocyte apoptosis leads to the development of DCM [79]. There are two central pathways involved in apoptosis, i.e., extrinsic pathway and intrinsic pathway. Several key pathways and potential signaling mechanisms during DCM progression have been summarized in Figure 4.

##### Extrinsic Pathway

The extrinsic apoptotic pathway involving caspase 8 is found to be associated with cardiomyocyte apoptosis leading to DCM in mice [79]. The extrinsic apoptosis pathway is mediated by a class of receptors called death receptors present on cardiomyocytes. These receptors include TNF-related apoptosis-inducing ligand (TRAIL) receptors, Fas receptors and TNF receptors [80]. These generic pathways become activated during the pathological progression of DCM.

##### Intrinsic Pathway

The intrinsic apoptosis pathway mediated by mitochondria dysfunction leads to cardiomyocyte apoptosis in DCM [81]. The intrinsic pathway is the integration of stress cues from the internal environment of the cells and can be triggered by different stimuli such as nutrient deficiency, radiation, drug, oxidative stress, irreparable DNA damage, and the misfolding of proteins. Mitochondrial and endoplasmic-reticulum stress has also been found to be involved in the apoptosis of cardiomyocytes in cardiovascular disease conditions [82].

#### 4.1.2. Fibrosis Pathways Associated with DCM

**Figure 5:**
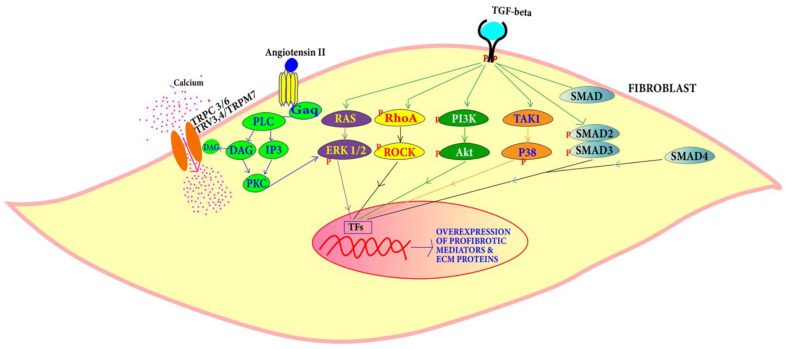
TGF-β can induce cardiac fibroblast proliferation through SMAD-dependent and SMAD-independent pathways, while proliferation through Ang-II is mediated by PLC, DAG, IP3 and PKC.

TGF-β and Ang-II pathways are known in the DCM condition to induce the proliferation of cardiac fibroblasts to myofibroblasts leading to fibrosis (Figure 5) [83,84,85,86,87].

*TGF-β Pathway:* TGF-β is a signaling molecule that is involved in a plethora of signaling pathways. TGF-β can change the expression of extracellular matrix components [88]. TGF-β levels are found to be high in DCM patients. The overexpression of TGF-β in mouse heart leads to myocardial fibrosis [83]. Canonically, TGF-β type II binding with its receptor causes TGF-β type I receptor stimulation which activates the SMAD complex. This heterotrimeric complex of SMAD 2, SMAD 3 along with SMAD 4 that is translocated in the nucleus, binds to a section called the SMAD binding elements (SBE) and promotes collagen deposition and fibrosis. SMAD-independent pathways involve PI3K/AKT and MAPK pathways and RHO/RAC1. High TGF-β expression triggers the differentiation of myofibroblasts and the deposition of matrix components associated with hypertrophic cardiomyopathy and DCM [83,84]. An isoform of TGF-β called TGF-β1 promotes cardiac apoptosis and hypertrophy and may be involved in cardiomyopathic pathology [85,86]. TGF-β binding to type II of TGF-β receptor activates TGF-β-activated kinase 1 (TAK1). TAK1 activation leads to the activation of p38 [89]. The action of TGF-β on Ras-Raf-MEK-ERK cascade causes the translocation of extracellular regulated protein kinases (ERK) and various transcription factor phosphorylation [90]. For example, activator protein 1 (*AP1*) gene is expressed to produce various ECM proteins. Along with regulating actin cytoskeleton, ROCK phosphorylate targets that are important for remodeling include myosin light chain (MLC), myosin-binding subunit (MBS) on MLC phosphatase (MLCP). Upregulation of ROCK leads to cardiac remodeling [91].

#### 4.1.3. Angiotensin Pathway

The renin angiotensin pathway has been revealed to be far more complex than was previously perceived. It is known to be involved in local transduction via the intracrine and paracrine or autocrine pathways as well as acting systemically. Mice develop DCM in response to angiotensin-II [87]. Angiotensin II (Ang II) can bind to two receptors: angiotensin type I (AT1) and angiotensin type II (AT2). The release of Ang II activates G protein-coupled receptors inducing the conversion of guanosine-5′-diphosphate (GTP) to guanosine-5′-triphosphate (GDP) via Gαq that interacts with phospholipase C (PLC). This stimulates diacylglycerol (DAG) and IP3 that activate the protein kinase C (PKC). GPCR activation also triggers Ca^2+^ release via DAG that is then converted to triacylglycerol (TAG) which controls Ca^2+^ influx into the fibroblasts from TRPC 3/6 TRV3,4/TRPM7 and regulates cardiac contractile adaptation [92]. PKC induces ERK1/2 leading to the activation of transcription factors that cause fibroblast proliferation into myofibroblasts.

## 5. Diagnosis of DCM

### 5.1. Clinical Investigation

Clinical investigation is the front-line diagnosis procedure. Many patients do not manifest diagnostic symptoms until they are at the advanced stage of heart failure [93]. Decreased cardiac output and arrhythmia are general symptoms of DCM but many individuals with DCM are asymptomatic. Family history plays a significant role in DCM [3]. The investigation of family history of disease includes type 2 diabetes, epilepsy, deafness and other metabolic disorders [94].

### 5.2. Electrocardiography (ECG)

In patients with DCM, the electrocardiograms are not generally normal and patients with extensive fibrosis in the left ventricle are observed with isolated T wave change to the septal Q wave [95]. Delay in atrioventricular conduction and a bundle branch block are also likely to be observed [95]. Due to atrial fibrillation, sinus tachycardia and supraventricular arrhythmia can be observed [96].

### 5.3. Echocardiography

Echocardiography is most essential and one of the gold-standard techniques in the diagnosis of DCM. The echo diagnostic criteria of DCM includes the left ventricle area >112% which indicates dilation of the left ventricle. A fraction shortening <25% indicates abnormal systolic function. A left ventricle area >117% is a criterion for screening familial DCM (Table 3) [97].

### 5.4. Magnetic Resonance Imaging (MRI)

MRI is useful in determining chamber dimensions, wall thickness and ventricle mass. MRI is also useful in the assessment of flow rate, abnormality in wall motion, edema, fibrosis and hyperemia [98].

### 5.5. Serological Test for Virus

Serological testing for virus by specific IgM antibody titer test can be useful in detecting viral myocarditis [99].

### 5.6. Endomyocardial Biopsy

This is the most definitive method for the diagnosis of DCM. An endomyocardial biopsy can diagnose patients who cannot be screened through other methods. Endomyocardial biopsy can detect various myocardial diseases [100]. However, this is an invasive, costly method warranting specialized expertise. Hence, it has limitations for screening in general population.

### 5.7. Clinical Genetics of DCM

Genetic testing is now widely used in the detection of DCM. Genetic testing is crucial in the case of asymptomatic patients for better management and early intervention of subjects. Family genetic history using pedigree analysis has remained a classical approach in the diagnosis of genetic DCM. Pedigree analysis can help in the diagnosis of mutation in family-specific genes by genetic testing. However, it has remained a challenge to ascertain the specific genes causing DCM. Moreover, more than 100 genes have been reported to be involved in the etiology of DCM [101]. Commercial laboratories are using a gene panel (~60 genes) to test the susceptibility of developing DCM in different populations. Recently, Jordan et al. [101] evaluated the potential of a large gene panel for DCM prediction and summarized 12 definitive (strongly associated, included in Table 2 and shown in Figure 2) genes to be exclusively associated with the DCM pathophysiology, not hypertrophic cardiomyopathy (HCM) or any other cardiomyopathy. The panel of these 12 genes should be evaluated across different populations to be implemented as a standard genetic test to screen for DCM patients [13,34].

### 5.8. Pathological Tests for DCM Diagnosis

Several pathological tests in isolation or in combination are performed in order to detect the clinical severity of DCM patients (Table 4) [22,47,77,78,102].

### 5.9. Protein Biomarkers of DCM

There are alterations in the protein content in the heart during the development of DCM. This alteration can be detected by proteomic techniques [103]. These alterations in the protein content indicate the progression of DCM. Various gene mutations work as genetic biomarkers for the diagnosis of DCM, in the same way that proteins are generally used as biomarkers of DCM.

#### 5.9.1. Brain Natriuretic Peptide (BNP)

Brain natriuretic peptide indicates stress on the ventricles. Brain natriuretic peptide levels predict DCM as well as heart failure. *BNP* levels accurately diagnose DCM and heart failure. Brain natriuretic peptide can also be used to assess the effectiveness of a heart therapy [104].

#### 5.9.2. ST2

ST2 is a cardiac biomarker, which is a member of the interleukin-1 receptor family. ST2 is released by cardiomyocytes in stress conditions. The presence of ST2 indicates tissue fibrosis, cardiac remodeling and abnormality in the heart. ST2 can diagnose a heart abnormality at an earlier stage than the brain natriuretic peptide [105].

#### 5.9.3. Troponins T, I

Troponins T and I are sensitive biomarkers of heart failure. A modest increase in the serum level of troponins T and I predict abnormality in the heart. Most of heart failure patients have elevated levels of high sensitivity troponin in the serum [106].

#### 5.9.4. Procollagen Type III

Collagen also works as heart failure biomarker. Elevated levels of collagen predict abnormality in the heart. Procollagen type III independently predicts progression of heart diseases [107].

#### 5.9.5. Matrix Metalloproteinase (MMP)

Matrix metalloproteinases work as biomarkers for DCM and heart failure. Elevated levels of matrix metalloproteinase predict cardiac dilation and a decrease in ejection fraction. Elevated levels of *MMP*-2, -7, -8, and -9 are found in children with DCM [108].

#### 5.9.6. Galectin-3

Under stressed conditions, activated macrophages produce galectin-3 protein. Galectin-3 protein predicts abnormality in the heart. Galectin-3 in combination with N-terminal brain natriuretic peptide (proBNP) is the best biomarker for the diagnosis of acute heart failure. It is also useful in assessing the effectiveness of a therapy in individuals with heart disease [109].

## 6. Treatment Strategies of DCM

There is no treatment available to permanently cure or ameliorate the DCM condition, but several treatment regimens are clinically practiced to restrict the progression of DCM and further reduce the symptoms. The current state-of-the-art therapeutic regimens described below aim to prevent heart failure and thromboembolism.

### 6.1. Angiotensin-Converting Enzyme (ACE) Inhibitors

In all etiologies of heart failure, the activation of the renin angiotensin aldosterone system (RAAS) is significant. To block this pathway, ACE inhibitors are used. These inhibitors are found to relieve dyspnea and reduce heart disease progression. ACE inhibitors work by preventing the formation of angiotensin II which is a vasoconstrictor that inhibits the hydrolysis of bradykinin, which is a vasodilator [110]. Thus, ACE inhibitors prevent blood load on blood vessels and decrease blood pressure. Commonly used ACE inhibitor drugs are Benazepril, Perindopril, Trandolapril, Zofenopril, and Ramipril. Adverse effects of ACE inhibitors are also observed in some patients [111]. Impairment in renal function is a major side effect, hypotension and cough are also adverse effects of the use of ACE inhibitors [111].

### 6.2. Diuretics

Diuretic drugs are useful in treating heart disease. Diuretics increase the excretion of water and salts from the body, which lowers the pumping load from the heart. These drugs work by inhibiting the antidiuretic hormone vasopressin and they work to reduce the blood pressure, swelling, and water buildup [112,113]. The side effects of diuretics include increased peeing, tiredness, weakness, muscle cramps, blurred vision, headache, increased sweating, and dehydration.

### 6.3. Angiotensin II (Ang-II) Receptor Antagonists

These are angiotensin II receptor blocking drugs. These drugs bind to angiotensin II receptors and actively inhibit their function. Thus, arteriolar contraction and sodium retention is prevented by angiotensin II receptor antagonists [114].

### 6.4. Beta Blockers

Beta-blockers are widely used to control heart disease. Beta-blockers are efficient in temporarily reducing blood pressure, angina, arrhythmia, anxiety, migraine, glaucoma, and overactive thyroid [115]. Beta blockers are useful in preventing heart attack [116]. Adrenaline and noradrenaline hormones are responsible for inducing the fight or flight response in the body in the presence of any danger. These hormones increase oxygen demand, heartbeat, arrhythmia, blood pressure, anxiety, sweating and palpitation in the body [116]. These hormones excessively increase the load on the heart. Beta blockers block the action of these hormones by competitively binding with the beta-adrenergic receptors of endogenous catecholamines epinephrine (adrenaline) and norepinephrine (noradrenaline) hormones. Β_1_-adrenergic receptors are found in the cells of the heart which are blocked by beta blockers [117].

### 6.5. Spironolactone

Spironolactones are used to lower blood pressure. Spironolactones are useful in preventing heart failure and in some cases, they are also useful in managing edema. Aldosterone is present in high amounts in DCM patients, retains sodium inside the body, excretes potassium and causes baroreceptor dysfunction [118]. Mineralocorticoid receptors in the distal convoluted tubule are competitively inhibited by spironolactones [118]. This inhibition increases water and sodium excretion and potassium retention [118].

### 6.6. SGLT2 Inhibitors (SGLT2i)

In the comorbid condition of diabetes mellitus and cardiovascular disease, SGLT2 inhibitors are quite useful. These molecular inhibitors of sodium-glucose-co-transporter 2 reduce the blood glucose level by promoting the urinary excretion of glucose. The unique mode of action of SGLT2 inhibitors results in glycosuria. SGLT2 inhibitors show diuretic and natriuretic actions. These actions help in decreasing blood pressure and plasma volume. A decrease in albuminuria and the glomerular infiltration rate is also associated with SGLT2 inhibitor treatment [119]. SGLT2 are becoming the primary choice for treatment in patients having diabetes with diabetic kidney disease (DKD) and cardiovascular diseases [120]. Empagliflozin, canagliflozin and dapagliflozin are available as SGLT2 inhibitor drugs used in clinical practice.

### 6.7. Potential Novel Treatments of DCM

#### 6.7.1. Cytokine Antagonists

Degraded myocardium, activated macrophages and T cells secrete tumor necrosis factor α (TNF-α) and other secreted proinflammatory cytokines to promote the progression of cardiomyopathy [121]. The pentoxifylline drug is a xanthine derivative which actively suppresses the production of TNF-α [122]. Endothelin peptides are produced in high amounts in DCM patients and these peptides contribute to vasoconstriction leading to increased blood pressure. Bosentan is an endothelin antagonist which helps in lowering the blood pressure [123].

#### 6.7.2. Anticoagulants

In patients with a history of thromboembolism, high ventricular dilation, and severe systolic dysfunction, anticoagulants are used in treatment [124]. Blood thinner drugs prevent stroke in people with severe heart disease. Warfarin is an anticoagulant drug that prevents blood coagulation by blocking the enzyme vitamin K epoxide reductase.

#### 6.7.3. Natriuretic Peptides

Atrial natriuretic peptides (*ANP*) are natural vasodilators and diuretics which are secreted by atrial myocytes in response to stretch/stress or other stimulations [125]. These may be useful in DCM. Tolerance against these peptides is gained in a short time so these cannot be administrated for long periods and research is needed to make drugs based on them for use in heart disease.

#### 6.7.4. Stem Cell Therapy

Stem cell therapy is an exciting newer approach studied for the treatment of DCM. Autologous bone marrow cell therapy for DCM in the short term (6 months) and long term (3 years) showed beneficial results [126]. In the future, stem cell therapy could be highly useful in the treatment of DCM.

#### 6.7.5. Clinical Trials

Drugs undergoing clinical trials for DCM in the United States are listed in Table 5.

### 6.8. Assisting Devices and Mechanical Support

#### 6.8.1. Partial Left Ventriculectomy (PLV)

PLV is used in the treatment of DCM. It reduces heart wall pressure by resection of a portion of the left ventricle which lowers cardiac volume [128]. PLV is used on patients who are ready to receive a heart transplant but are unable to receive it [129].

#### 6.8.2. Left Ventricular Assist Devices (LVADs)

LVADs are used in end stage heart failure patients. LVADs are used as a bridge to heart transplantation. Advanced LVADs can be used as a replacement of transplant [130].

#### 6.8.3. Multisite Ventricular Pacing

Patients with DCM have abnormal left ventricle function. A delay in conduction of the left ventricle is also associated in the prolongation of conduction of the atrioventricle. The synchrony in the function is restored by dual-chamber pacing [131]. Single ventricular pacing is not considered sufficient, so biventricular pacing is used. Pacing is used in patients having a QRS duration of more than 150 ms [131].

## 7. Epidemiological Studies in India on DCM

Several candidate-gene-based epidemiological studies have been performed in India. Most importantly, Dhandapani et al., in 2009, published a work highlighting the role of defects (25 bp deletion) in the gene myosin binding protein C in the heart represented as *MYBPC3*, in a South Asian population with heritable conditions of cardiomyopathies such as DCM or HCM and contributing to heart failure. The sample collection for the cardiomyopathy investigation was carried out in hospitals in Madurai, Trivandrum, Hyderabad, Kozhikode, Chandigarh, and Mumbai [132]. Das et al. and Kothari et al. examined DCM clinically and genetically, focusing on the Indian adult and child population, respectively [133,134,135]. The samples were collected under the “Epidemiology of Cardiomyopathy DCM study” with 80 people out of the cohort being DCM patients. Forty percent were the result of the familial type of DCM with the majority showing autosomal dominant inheritance and 48% were sporadic. Rai et al.’s work on the clinical and genetic profiling of cardiomyopathy (idiopathic restrictive and hypertrophy) investigated sarcomere-associated gene mutation effects [136]. They found the contribution of *TNN13* and a novel *MYH7* (beta myosin heavy chain) mutation in cardiomyopathy development and a new mutation found to be associated with DCM induction [136]. Another study on *MYH7* genetic variations carried out by Tanjore et al. analyzed the mechanism behind DCM-phenotype development. The samples belonged to a hospital in Hyderabad and common SNPs were found in exons 7, 12, 19 and 20 in DCM and HCM conditions. These SNPs when present in homozygous conditions gave rise to DCM [137]. Similarly, in another work, they aimed to reveal ACE indel (I/D) mutation-associated cardiomyopathic development in the Indian population showing reduced LVEF in DCM patients, which pointed towards the influence of ID genotype on cardiomyopathic phenotype [138]. A first case study presented by Jadhav et al. unravels a unique case of Emery-Dreifuss muscular dystrophy (EDMD2) which is autosomal dominant and associated with *LMNA* mutations appearing in a familial DCM patient [139]. Endothelial nitric oxide synthase gene (*NOS3*) with three functional polymorphisms resulted in its variable expression. This led to DCM progression due to oxidative stress from the imbalanced production of nitric oxide which causes impairment in the contractile property of cardiomyocytes [140]. The role of the endothelin 1 gene (*EDN1*) was investigated by Matsa et al. in the manifestation of DCM in 115 DCM patients from a hospital in Hyderabad, using PCR-based followed by single-stranded confirmation polymorphism analysis along with the commercial screening insertion variation (+138 A) in a heterozygotic state leading to a four-fold increased risk of DCM [141]. Troponin T (c*TNNT*)-related genetic variations were studied by Rani et al. in 147 DCM patients of south-Indian origin. Exon analysis revealed a novel mutation R144W in the tropomyosin binding domain that was responsible for distorting the protein structure and function by changing the residue charge [142]. A paper presented by Deshmukh et al. from Uttar Pradesh, where DCM is sparsely studied, revealed different causes and etiologies of this condition in 100 patients over a four-year period [143]. Similarly, a cross-sectional study over a period of three years was conducted in Gujarat with 180 DCM patients. This descriptive study calculated the etiological factors contributing to DCM such as diabetes, alcohol, nutritional, post-partum, etc. [144]. A significant DCM cross-sectional study from Ranchi by Saha et al. of 30 DCM cases provided prevalence data of DCM [145]. Ushasree et al. explored the genetic heterogeneity and epidemiological factors associated with disease expression in the Indian context [146]. Another rare account of DCM from the north of the country came from Shairgojri et al., providing a comprehensive view of the condition in children. From the analysis of samples collected from a pediatric hospital in Srinagar, a 51.35% DCM pathology was found, arising due to different causes [147]. Recently, a DCM study from Assam provided a comprehensive perspective on the cardiac condition. An observational study characterizing DCM prevalence based on its causes was carried out in North East India. A cadaveric study of DCM patients to measure the anatomical alterations of the cardiac wall in samples from North India, giving significant insight into the morphological changes undergone by the heart muscle, was made by Bose et al. [148]. A study showing the effects of hypertension on DCM patients was performed by Balije et al. that would help in the diagnosis of DCM by evaluating blood pressure response [149]. Ahmad et al. presented a clinical and etiological profile of DCM patients from 55 samples and evaluated the echocardiographic and electrocardiographic profiles which would prove to be highly significant in diagnosis of the condition [150].

Table 6 (supplementary Figure S1) summarizes these independent DCM studies carried out across different states of India. It can be clearly observed that most of these studies are from the candidate genomic perspective. There has been major progress in DCM study in the Indian context and genomics has disclosed major secrets about the said pathology. However, recently it has been shown that approximately only 40% of the DCM cases stem from a genetic defect or mutation [13]. This implies that the genetic variations could explain only a certain number of cases of DCM pathological progression in patients.

## 8. Limitations of Candidate-Gene Based Studies: Newer System-Level OMICS Approaches to the Rescue

So far in India, DCM has been looked at from a candidate-gene perspective. Genetic evidence from Indian populations points to the fact that the majority of the descendants of ethnolinguistic groups include a blend of various divergent ancestral natives such as those of North Indian lineage sharing kinship with West Eurasians, and Native Andaman Islanders being ancestral to Southern Indians [154,155]. This admixture of genes and genetic diversity contributes to many variations in genome-reliant studies. Thus next-generation omics-based methods focusing on “proteins” and “lipids” may shed critical insights into the pathological progression of DCM as well as the identification of newer potential biomarker panels with better prognostic value. In recent years, proteomics has emerged to be a highly revelatory and effective approach in cardiovascular disease research [156]. It makes use of high throughput and sensitive techniques such as mass spectrometry for biomarker investigation, pathway elucidation, and post-translation modification studies [88]. Additionally, lipidomics provides a comprehensive view of the metabolic rewiring of individuals progressing towards a disease state [157,158]. It is now time to exploit the collaboration of the latest technological advancements and medical data to broaden our understanding of pathology. This would provide an integrative view instead of a reductionist approach to the disease. This can be achieved by incorporating systems biology techniques in our research that integrate molecular information from different hierarchies to interpret into models that demonstrate complex biological processes [159]. Systems biology and proteomic analysis have given a new platform for biomarker studies and extensive research is being carried out to discover novel biomarkers. These biomolecules would help assess the progression of CVD just by the quantitative analysis of components of plasma. Recently, Izquierdo et al. performed a biomarker study by proteomic analysis using 2D-DIGE and mass spectrometry in DCM patients carrying the LMNA pathological gene mutation [160]. Another interesting study systematically characterized the circulating proteins at different stages of heart failure by employing aptamer-based proteomic profiling [161]. These significant studies will be corroborated with the novel proteomic and lipidomic candidate biomarkers identified in Indian DCM patients.

## 9. Systems Biology Approach for Better Diagnosis of DCM

Proteomic techniques combined with lipidomic concept application to study heart disease can give us new insights into the biomolecular mechanisms and changes undergone by tissue during disease progression. Our next focus in this context is to probe mass-spectrometry-based “clinical-omics” studies of DCM patients from India. This initiative has been recently funded by the Indian Council of Medical Research (ICMR). We aim to develop a noninvasive diagnostic multimarker panel (proteomic and lipidomic) that would be indicative of DCM status in Indian patients.

## Figures and Tables

**Table 1:** Symptoms of DCM in adults and children [7,8,9,10].

Symptoms of DCM in Adults	Symptoms of DCM in Children/Newborns
Dyspnea and fatigue	Poor appetite/Problems in feeding
Dizziness	Poor growth
Syncope	Excessive sweating during feeding
Edema	Sweating while doing any activity
Palpitations	Palpitations
Controlled weight loss/gain	Fast breathing/difficult breathing
Excessive Sweating	Edema
Abdominal discomfort	
Nausea	
Anorexia	
Cachexia	

**Table 2:** Mutations in cytoskeletal and sarcomeric protein-coding genes are majorly associated with DCM [31,32,33,34].

Cytoskeletal and Sarcomeric Genes	
Titin (*TTN*)	Cardiac actin alpha
Desmin (*DES*)	Troponin T2, I3, C1 (*TNNT2*, *TNNI3*, *TNNC1*)
Lamin A/C (*LMNA*)	β-myosin heavy chain (*MHY7*)
α-actinin-2 (*ACTN2*)	Tropomyosin-1 (*TPM1*)
Actin binding LIM domain protein (*ABLIM1*)	Phospholamban (*PLN*)
Nebulette (*NEBL*)	Myosin binding protein C (*MYBPC3*)
Myopalladin (*MYPN*)	Sodium channel protein type 5 subunit alpha (*SCN5A*)
Filamin C (*FLNC*)	BCL2-associated athanogene 3 (*BAG3*)
δ sarcoglycan (*SGCD*)	
Vinculin (*VCL*)	
Z band alternatively spliced PDZ domain protein (*ZASP*)	
Dystrophin gene (*DMD*)	

**Table 3:** Echocardiographic diagnosis of dilated cardiomyopathy [Adapted with permission from Mathew et al.] [97].

View	Measurement	Description
Plax (parasternal long axis) M-mode	LVIDd (left ventricular internal dimension in diastole). LVPWd (left ventricular posterior (inferolateral) wall thickness in diastole). IVSd (interventricular septal thickness diastole).	Left ventricle cavity size >112% (2 S.D.) based on surface area and age. Left ventricle cavity size >117% is specific criterion in screening.
	Fraction shortening	FS < 25%
	Mitral valve E-septal separation	Normal range—0–5.3 mm. Reduced systolic function is indicated by value above 7 mm.
PLAX (parasternal long axis) color flow Doppler	Mitral regurgitation	1. Leaflets have symmetric tenting. 2. Coaptation zone of leaflet is reduced. 3. Central jet of MR. 4. Coaptation depth is increased. 5. Tenting area is increased. 6. Mitral valve annulus is dilated.
A4C (apical four chamber) PW	Diastolic Dysfunction	Diastolic dysfunction is present in patients with EF < 45%.
A4C 2D	Left ventricle ejection fraction	EF < 45% indicates DCM
	Sphericity index	Normal > 1.5, decrease in sphericity index value to near 1 indicates DCM [97].

**Table 4:** Pathological tests for DCM diagnosis [22,47,77,78,102].

Pathological Tests	Other Tests in Specific Indications
Erythrocyte sedimentation rate (ESR)	Coronary angiography
Viral serology	Blood content tests—carnitine, pyruvate, lactate, autoantibodies, selenium, acylcarnitine profile and drug screening.
Creatine kinase (CK)	Red cell transketolase (beri beri)
Liver function tests	Urine
Renal function	Enteroviruses test
Serum ferritin/iron/transferring	Infective screening (HIV/hepatitis C)
Thyroid function tests	Organic acid/amino acids

**Table 5:** Description of drugs for dilated cardiomyopathy undergoing clinical trials in the United States [127].

Drug	Title of Project	Description	Disease or Condition	Location
**ARRY-371797,** (**p38 inhibitor**)	A rollover study of ARRY-371797 in patients with LMNA-related dilated cardiomyopathy	Assessment of effectiveness of drug ARRY-371797 is being investigated in this clinical trial.	LMNA-related dilated cardiomyopathy	University of Colorado Aurora, Colorado, United States Johns Hopkins University Baltimore, Maryland, United States
**ARRY-371797**	A study of ARRY-371797 in patients with symptomatic dilated cardiomyopathy due to a lamin A/C gene mutation	This study is a placebo controlled, dose-dependent efficacy assessment of ARRY-371797 drug on LMNA gene mutation dilated cardiomyopathy patients.	Lamin A/C gene mutation dilated cardiomyopathy	Pfizer Investigational Site Birmingham, Alabama, United States. CB Flock Research Corporation Mobile, Alabama, United States, and 64 more.
**Ivabradine**	Pulse reduction on beta-blocker and Ivabradine therapy	Ivabradine improves ejection fraction by reducing heart rate independently from beta-blockade.	Dilated cardiomyopathy ventricular remodeling electrical remodeling	University of Colorado Anschutz Medical Campus Aurora, Colorado, United States The Ohio State University Wexner Medical Center Columbus, Ohio, United States
**Ifetroban**	Oral Ifetroban in subjects with Duchenne muscular dystrophy (DMD)	X-linked Duchenne muscular dystrophy (DMD) is a fatal genetic disorder. This lacks effective treatment therapy. Ifetroban is assessed in this study for the treatment of DMD.	Duchenne muscular dystrophy cardiomyopathy dilated cardiomyopathy	Mattel Children’s Hospital Los Angeles, California, United States and 3 more.

**Table 6:** Summary of DCM studies performed across different states of India.

Age Group	Male or Female (%)	Number of Cardiomyopathy Patients	Study Description	Sample Collection	Focus Population	Reference
41.7 ± 16.5	38.70% males	80 DCM	40% familial, 48% sporadic. LMNA (c. 639 + G > C) associated novel splice site and MYH7 (c. 2769 > T) rare varient found.	EPOCH-D Study, AIIMS, New Delhi	General [151]	Das et al., 2015
15–67	35.3% males	61 DCM	61% DCM patients with *MYH7* gene (exon 8–24) mutation with a novel p.Gly377Ser mutation found.	Nehru Hospital, Chandigarh	Asian Indians [136]	Rai et al., 2009
45.41 ± 14.35	54% males	51 DCM	Influence of D allele in DCM development associated with ACE I/D	PGIMER, Chandigarh, Rajaji Government hospital, Madurai, and, Sri Chitra Tirunal Institute of Medical Sciences and Technology, Trivandrum	General [138]	Rai et al., 2008
Juvenile–Adult	Undefined	97 DCM	Common exon link of *MYH7* gene IN DCM and HCM. Homozygous condition develops DCM.	CARE Hospitals, Mahavir hospitals and Niloufer hospitals, Hyderabad	General [137]	Tanjore et al., 2010
53 year old	1 male	DCM Case Study	Familial DCM along with Emery-Dreifuss myopathy associated with mutation in *LMNA* gene.	Lourdes Heart Institute and Neuro Center, Kochi, Kerala	Kerala [139]	Jadhav et al., 2012 [22]
>25 years old	Undefined	115 DCM	Influence of endothelial *NOS3* gene in DCM manifestation by oxidative stress production.	CARE hospitals, Krishna Institute of Medical Sciences (KIMS) and Niloufer hospital for Children, Hyderabad, India	General [140]	Matsa et al., 2013
33.2± 16.1	70% male 30% females	115 DCM	Screening of *EDN1* gene and two rare genetic variations found. Insertion variations (+138 A) corresponding to heterozygotic condition leads to 4-fold increased risk of DCM.	CARE hospitals, Krishna Institute of Medical Sciences (KIMS) and Niloufer Hospital for Children, Hyderabad, India	General [141]	Matsa et al., 2014
NA	NA	147 DCM	Role of novel mutation R144W in Toponin T binding domain found by overall exon analysis in DCM development.	NA	South Indian [142]	Rani et al., 2014
2.9 ± 3.07	50 males	80 DCM	Pediatric DCM was explored in this observational study.		General [135]	Kothari et al., 2003
Above 60	66.6% Males 33.4% females	100 DCM	4-year study of DCM charecterization based on cause. DCM in peripartum females accounted for 9%, DCM in smokers was 65%, 30% alcoholic DCM.	M.M.C. Muzaffarnagar, U.P, & SIMS, Hapur, UP	Western Uttar Pradesh [143]	Deshmukh et al., 2011
Above 60	56.6% males 43.25% females	30 DCM	Cross-sectional study of DCM over 1 year period. Ischemic DCM 33.3%+ diabetic cardiomyopathy (23.3%) peripartum cardiomyopathy (16.6%) 6.6 Alcoholic and miscellaneous DCM	Rajendra Institute of Medical Sciences, Ranchi.	India General [145]	Saha et al., 2018
Above 13	43% male, 56.66% females	180 DCM	3-year DCM etiology study. Diabetes 13.33% alcohol 23.33% postpartum 15.00% idiopathic 30.00% inflammation 03.33% nutritional 6.66% multifactorial 06.66%	GMERS General Hospital, Gotri, Vadodara.	Indian General [144]	Rana et al., 2015
6 months–Adult	62.61% males 37.38% females	107 DCM	Parental consanguinity 22.42% alcoholic/smoking DCM	KIMS and Niloufer Hospital for children, Hyderabad	Indian General [146]	Ushasree et al., 2009
1 month–18	52.65% males 47.6% female	19 DCM	Hospital based observational profile of DCM in children. Study period of two and half years, charecterization made on symptoms, gender preponderance parental consanguinity and myocarditis.	Post Graduate Department of Pediatrics Government Medical College, Srinagar	North (Kashmir) [147]	Shairgojri et al., 2021
18–80	61.29% males 38.71% females	31 DCM	Observational study characterizing DCM on the basis of its causes. Alcoholic DCM 23.33% + viral DCM 03.33% peripartum cardiomyopathy 22.58% idiopathic DCM 41.93% familial DCM 9.6%	Jorhat Medical College and Hospital, Jorhat, Assam	North East India [152]	Sonowal et al., 2014
15–75	18.3% females	60 DCM	An analytical and observational study showed 35 patients DCM with hypertension and 25 DCM patients without hypertension	Cardiology, GSL Medical College and Hospital, Rajahmundry India	Andhra Pradesh [149]	Balije et al., 2016
50 ± 15	65.45% males 34% females	55 DCM	1-year clinical and incidence profiling of idiopathic DCM	JN Medical College, Aligarh	General [150]	Ahmad et al., 2005
All	61.4% males 38.6% females	70 DCM	Pilot study to determine different demographic parameters in DCM patients. 27% alcoholic 46% smokers in the cohort	Tertiary Medical College of Eastern India	Eastern India [153]	Paul et al., 2014
10–70 years	NA	10 DCM	Measuring the gross morphological changes in heart wall of DCM patients	Institute of Medical Sciences, BHU, Varanasi (U.P)	North Indian [148]	Prasenjit Bose et al., 2017

## Data Availability

Not applicable.

## Supplementary Materials

The following are available online at https://www.mdpi.com/article/10.3390/jcdd9060174/s1, Figure S1: Representative map of India highlighting different studies performed on DCM across the country.
