# Supplementary material for: A Comprehensive Outlook on Dilated Cardiomyopathy (DCM): State-Of-The-Art Developments with Special Emphasis on OMICS-Based Approaches
Source: J Cardiovasc Dev Dis. 2022 Jun 1;9(6):174. doi: 10.3390/jcdd9060174 (PMC9225617; doi:10.3390/jcdd9060174)
Supplement: Supplementary file 1 [file jcdd-09-00174-s001.zip › jcdd-1522940-supplementary.pdf]

Journal of
Cardiovascular Development and Disease

MDPI

Review

A comprehensive outlook on Dilated Cardiomyopathy (DCM): State-of-the-art developments with special emphasis on OMICS based approaches

Vivek Sarohi[1,2], Shriya Srivastava[1], Trayambak Basak*[1,2]

Supplementary Information:

Supplementary Figure S1: Representative map of India highlighting different studies performed on DCM across the country.

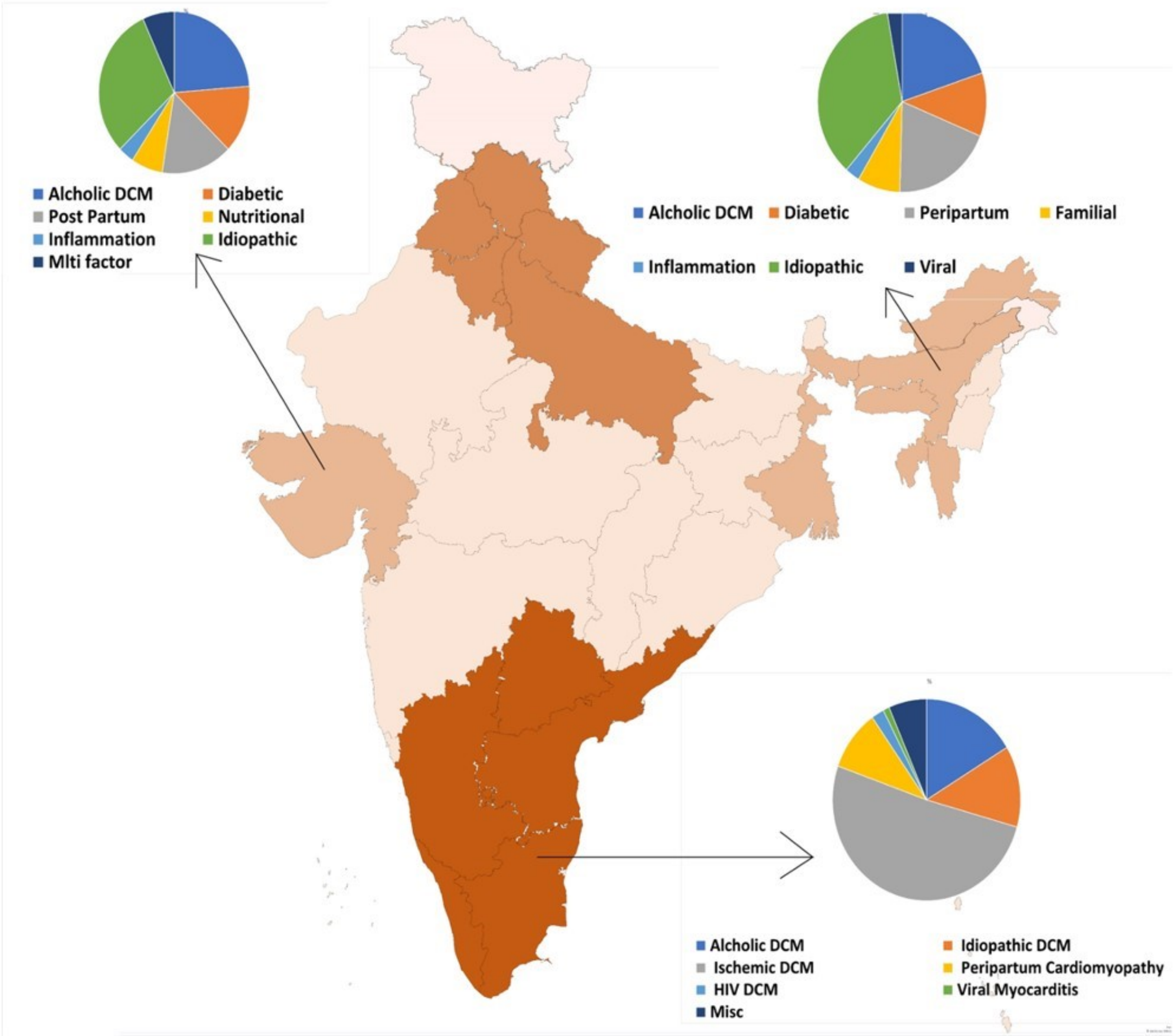

Figure S1. Representative map of India highlighting different studies performed on DCM across the country.
